# Role of Sodium-Glucose Co-Transporter 2 Inhibitors in Chronic Kidney Disease, Congestive Heart Failure and Stroke—A Review and Clinical Guide for Healthcare Professionals

**DOI:** 10.3390/jcm12196202

**Published:** 2023-09-26

**Authors:** Prabhat Singh, Lokesh Goyal, Deobrat C. Mallick, Salim R. Surani, Kanica Yashi

**Affiliations:** 1 Department of Nephrology, Kidney Specialist of South Texas, 1521 S Staples St., Corpus Christi, TX 78403, USA; 2 Department of Internal Medicine, Christus Spohn Hospital, 600 Elizabeth St., Corpus Christi, TX 78403, USA; 3 Department of Pulmonary Medicine, Texas A&M University, 400 Bizzell St., College Station, TX 77843, USA; 4 Department of Internal Medicine, Bassett Healthcare Network, Cooperstown, NY 13326, USA

**Keywords:** diabetic kidney disease, nephropathy, SGLT-2 inhibitor, stroke, CHF, MRA, flozins

## Abstract

Diabetic kidney disease (DKD) causes a progressive decline in renal function, leading to end-stage kidney disease (ESKD), and increases the likelihood of cardiovascular events and mortality. The recent introduction of the sodium-glucose co-transporter 2 (SGLT-2) inhibitor has been a game changer in managing chronic kidney disease (CKD) and congestive heart failure (CHF). These agents not only slow down the progression of kidney disease but also have cardioprotective benefits, including for patients with congestive heart failure and atherosclerotic cardiovascular disease. Some evidence suggests that they can decrease the risk of stroke as well. This review aims to provide a comprehensive overview of the role of SGLT-2 inhibitors in CKD and CHF and their efficacy in stroke prevention. This review includes a comparison with glucagon-like peptide-1 (GLP-1) agonist and finerenone; focuses on safety data, the potential benefits beyond glycemic control, and a review of significant trials; and provides guidance in clinical practice.

## 1. Introduction

Diabetic kidney disease is the most common type of chronic kidney disease (CKD) and end-stage kidney disease (ESKD), with substantial morbidity and mortality worldwide [1,2]. Chronic kidney disease and congestive heart failure frequently coexist in patients with underlying diabetes mellitus (DM). The association between diabetes mellitus and heart failure (HF) has been well documented. Chronic kidney disease is considered an independent risk factor for cardiovascular disease, and the likelihood of cardiovascular mortality increases with a decline in renal function [3].

Traditional medical management for CKD and CHF includes blood pressure control, tight glycemic management, and renin–angiotensin–aldosterone system (RAAS) blockade. In practice, angiotensin-converting enzyme inhibitors (ACE inhibitors) or angiotensin receptor blockers (ARBs) are the first line of agents in patients with hypertension and DKD, even in the absence of proteinuria. ACE inhibitors/ARBs have been shown to decrease the risk of progression of CKD in patients with proteinuria, although these agents have not been shown to reduce the progression to ESKD in nonproteinuric patients [4,5,6].

The introduction of SGLT-2 inhibitors has radically changed the therapeutic landscape for CKD and CHF patients. This has added new opportunities for improving outcomes in these patients. SGLT-2 inhibitors were initially developed as glucose-lowering medication. Still, during the earlier cardiovascular outcome trials (CVOTs), it became clear that there is a significant advantage for kidney protection and cardioprotection.

Currently, four SGLT-2i agents are available in North America: canagliflozin, dapagliflozin, empagliflozin, and ertugliflozin, whereas the fifth agent, sotagliflozin, is a combined SGLT-1 and SGLT-2 inhibitor that the FDA recently approved in May 2023 for use in the USA.

## 2. Mechanism of Action

SGLT-2 inhibitors work by inhibiting the SGLT-2 channel found in the proximal convoluted tubules. This channel is primarily responsible for absorbing most of the filtered glucose, which is >90%. This leads to glucosuria, which is more pronounced in individuals with diabetes than in non-diabetic individuals. The extent of glucose reduction is influenced by kidney function, and it declines when the eGFR falls below 60 mL/min per 1.73 m^2^. This effect is almost nonexistent with an eGFR below 30 mL/min per 1.73 m^2^ [7].

**Figure 1:**
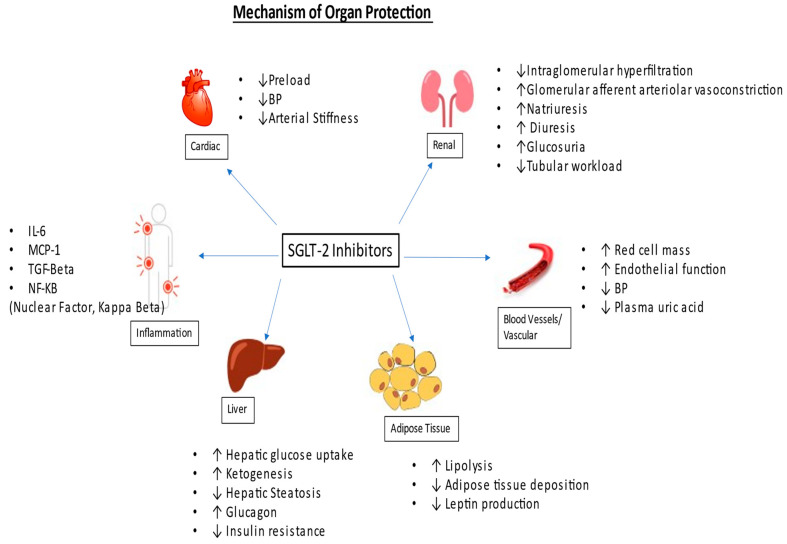
Mechanism of organ protection.

The renoprotective benefits of SGLT-2 inhibitors are independent of the glycosuria effect. It is proposed that SGLT-2 inhibitors reduce sodium reabsorption, leading to increased sodium delivery to the macula densa, which causes negative feedback through the tubuloglomerular feedback mechanism. This results in decreased intraglomerular pressure, causing lowered glomerular hyperfiltration [8]. Refer to Figure 1.

It has also been proposed that SGLT-2 inhibitors exert renal protection by improving tubular hypoxia by blocking proximal tubular reabsorption of glucose, reducing the workload of the proximal tubules [9]. Chronic tubular hypoxia has been attributed to the progression of underlying chronic kidney disease in patients with diabetes mellitus [10].

Beyond glucosuria, they also contribute to modest bp control, kaliuresis, and reduction in oxidative stress, as well as reducing markers of inflammation and fibrosis such as interleukin-6, monocyte chemoattractant protein 1 (MCP-1), nuclear factor-kB, urinary IL-6 as well as serum tumor necrosis factor receptor 1 (TNFR1) [11,12].

These kidney protective benefits lead directly to an improved metabolic profile and indirectly to beneficial effects on cardiovascular disease. Cardioprotective benefits include a decline in arterial stiffness and improvement in endothelial function. SGLT-2 inhibitors also cause diuresis and natriuresis, which may cause a reduction in preload [13,14].

## 3. Effectiveness

### 3.1. Effectiveness in CKD

Initial cardiovascular outcome trials (CVOTs), including EMPA-REG OUTCOME and CANVAS, were the first to demonstrate the kidney benefit as a secondary outcome. Subsequently, CREDENCE, DAPA-CKD, and EMPA-KIDNEY are the major randomized clinical trials that have solidified prior findings that SGLT-2 inhibitors are effective in slowing the progression to ESKD, reducing albuminuria, and preventing adverse outcomes, including the need for dialysis and cardiovascular mortality. All three trials were terminated early due to strong evidence of efficacy.

The CREDENCE trial included patients with an eGFR 30–90 mL/min/1.73 m^2^ and albuminuria (microalbumin-to-creatinine ratio (ACR) of 300–5000 mg/g) and 3.9% of patients had an eGFR less than 30 mL/min per 1.73 m^2^. It showed that canagliflozin at a dose of 100 mg once daily was effective in reducing the incidence of ESKD, heart failure hospitalization, all-cause mortality, and cardiovascular mortality, as well as doubling serum creatinine by approximately 30–35% [15]. In a separate subgroup analysis of patients with an eGFR less than 30 mL/min per 1.73 m^2^, it was found that it was equally effective in slowing down the progression of kidney disease as compared to patients with an eGFR greater than 30 mL/min/1.73 m^2^ [16].

DAPA-CKD included patients with an eGFR of 25 to 75 mL/min per 1.73 m^2^, proteinuria of 200 to 5000 mg/g, and one-third of the participants in this study had nondiabetic CKD. This trial showed that dapagliflozin 10 mg once daily was able to reduce the incidence of ESKD, all-cause mortality, and cardiac mortality by approximately 35–45%, doubling serum creatinine. The DAPA-CKD trial demonstrated that the protective benefit increases with an increasing hemoglobin A1c level as well as with increased proteinuria and decreased eGFR [17,18].

The EMPA-KIDNEY trial is the most recent, published in November 2022, which included patients with an eGFR of 20 to 44 mL/min/1.73 m^2^ (regardless of albuminuria) or an eGFR of 45 to 89 mL/min/1.73 m^2^ (if albuminuria > 200 mg/g). This was the first trial that included patients with no albuminuria; most patients in this trial (54%) had known chronic diabetic kidney disease. It demonstrated that empagliflozin 10 mg daily reduces the incidence of ESKD, cardiovascular mortality, and all-cause mortality by 25–30%, doubling serum creatinine. The most significant benefit was seen in patients with albuminuria ≥ 300 mg/g and less effect in patients with lower levels of proteinuria [19]. In this trial with a subgroup of normal albuminuria, the primary endpoint showed no improvement. It is unclear whether this was a false negative or a true negative result. One reason could be that this study was terminated early, after two years, so maybe the duration was not sufficiently long enough to see a difference. However, more studies are needed to evaluate the effect of SGLT-2 inhibitors in patients with normal albuminuria. (Table 1).

### 3.2. Effectiveness in CHF

The DAPA-HF, EMPEROR-Reduced, and EMPEROR-Preserved trials showed improvement in cardiovascular outcomes and a reduction in death and heart failure hospitalizations [20,21,22].

The DAPA-HF and EMPEROR-Reduced trials focused on patients with congestive heart failure with reduced left ventricular ejection fraction, which was at or below 40%. On the other hand, the EMPEROR-Preserved trial involved patients with a left ventricular ejection fraction greater than 40% [20,21,22].

The DAPA-HF trial included patients with chronic heart failure with LVEF < 40% and an eGFR ≥ 30 mL/min per 1.73 m^2^, and it showed significant improvements in composite primary outcomes, including reducing cardiovascular death and heart failure hospitalization by 24% [20]. The DAPA-HF trial indicated that dapagliflozin may prevent diabetes, and this study showed a decrease in the incidence of new diabetes mellitus [21].

The EMPEROR-Reduced trial enrolled patients with an eGFR >20 mL/min per 1.73 m and lower mean LVEF of up to 27% compared to up to 31% in the DAPA-HF trial. Empagliflozin reduced the combined risk of primary outcomes, including cardiovascular death or heart failure hospitalization, in patients with or without diabetes by 22% [22].

The EMPEROR-Preserved clinical trial was groundbreaking as it included patients with heart failure, regardless of whether they had mildly reduced ejection fraction or preserved ejection fraction or whether they had diabetes or not. Empagliflozin decreased the incidence of heart failure hospitalization and cardiovascular death by approximately 20%. Although, in this study, 23% of patients discontinued treatment, which was on the higher side, this outcome was similar in other treatment groups as well [23].

Similarly, the DELIVER trial was published in September 2022 and included patients with LVEF >40% with medical therapy. In this study, dapagliflozin showed a reduction in the primary composite endpoint by 18% [24].

### 3.3. Effectiveness in Stroke

SGLT-2 inhibitors have been very successful in improving outcomes in chronic kidney disease and cardiovascular disease. However, when it comes to preventing strokes, the available data are not yet as compelling.

Chronic kidney disease frequently coexists with other complex medical problems, including cardiovascular disease and stroke. Many experts believe that chronic kidney disease is an independent risk factor for stroke, and chronic kidney disease is present in over 40% of patients with stroke [25]

Stroke and chronic kidney disease have many risk factors in common, such as hypertension, diabetes mellitus, and atrial fibrillation. In the DECLARE-TIMI 58 trial, dapagliflozin was shown to decrease the incidence of atrial fibrillation [26].

In theory, one would believe that as SGLT-2 inhibitors improved outcomes in diabetes, kidney disease, and cardiovascular disease, they would also reduce the risk of stroke, but that has not been the case so far.

Multiple meta-analysis trials from recent cardiovascular outcome trials (CVOTs) showed no effect of SGLT-2 inhibitors in preventing strokes [27]. A meta-analysis of the credence trial showed that the SGLT-2 inhibitor showed no overall positive impact on total stroke prevention but had a slight benefit in terms of preventing hemorrhagic stroke [28].

However, most recently, in May 2023, an observational study from Taiwan published in the *Journal of the American Heart Association* suggested that an SGLT-2 inhibitor can decrease the incidence of new stroke by 20% in patients with atrial fibrillation [29]. This was an observational study to evaluate the impact of SGLT-2 inhibitors in preventing embolic stroke in patients with atrial fibrillation and diabetes mellitus. One of the drawbacks of this study was that it was not a randomized control trial. Nonetheless, this brings a ray of hope regarding stroke outcomes and provides direction for further studies.

#### 3.3.1. Safety Profile and Adverse Effects

Diabetic Ketoacidosis (DKA): DKA is a serious, life-threatening complication in patients with DM. SGLT-2 inhibitors can cause euglycemic diabetic ketoacidosis in patients with type 1 diabetes, but it is less common in Type-2 DM [30]. A recent meta-analysis in patients with Type-2 DM showed a slight increase in the relative risk of DKA. However, the absolute occurrence of DKA was low, with only around 0.2 events occurring per 1000 patient-years in the placebo group. In non-diabetic patients, the risk of DKA was nearly nonexistent, with only one episode noted during the following 30,000 patient-years [30].

In clinical practice, it is essential to identify patients with high risk for DKA, which includes recent surgery, starvation, acute illness, alcohol abuse, and those on high doses of insulin. Patients should be educated about the “sick day” plan to reduce the risk of DKA. This includes holding off on the SGLT-2 inhibitor for 2–3 days before surgery and temporary discontinuation during episodes of sickness or poor oral intake.

#### 3.3.2. Genital Mycotic Infections

It has been postulated that SGLT-2 inhibitors can increase the risk of urogenital infections due to glucosuria. SGLT-2 inhibitors can increase the risk of urogenital infection, such as vulvovaginal candidiasis, by 2–4 times, especially in female patients. In rare cases, SGLT-2 inhibitors are also associated with Fournier’s gangrene [31].

Patients must be educated and counseled on maintaining daily hygiene and monitoring for signs of early infection. Consider topical or oral antifungal therapy rather than discontinuation of the SGLT-2 inhibitor.

#### 3.3.3. Urinary Tract Infection (UTI)

A recent meta-analysis found that there is no increased incidence of urinary tract infections (UTIs) with SGLT-2 inhibitors as compared to other agents [32,33].

Expert consensuses suggest continuing SGLT-2 inhibitors in patients with recurrent urinary tract infections if there is an indication and exercising caution in those with a history of complicated recurrent urinary tract infections as well as chronic indwelling Foley catheters, but this is not an absolute contraindication.

#### 3.3.4. Lower-Limb Amputation/Fractures

The CANVAS trial indicated that there was a doubling of the risk of lower-limb amputation in the canagliflozin groups, and this mainly involved toe or metatarsal amputation and fractures [34].

However, subsequent major clinical trials, including CREDENCE, reported no difference in the risk of amputation between SGLT-2 inhibitors and amputation or fracture. As a result, in 2020, the FDA removed its black box warning for canagliflozin and amputation risk [30].

In practice, clinicians are advised to educate patients to perform frequent foot examinations, timely intervention, and referral to evaluate for peripheral vascular disease.

#### 3.3.5. Volume Depletion

Due to their natriuretic effects, SGLT-2 inhibitors can increase the risk of volume depletion, especially if combined with diuretics. The DAPA-HF trial reported an increased incidence of volume depletion, especially in patients who were taking furosemide or its equivalent dose ≥ 40 mg daily. In this trial, the placebo group had a 6.8% incidence of volume depletion, whereas it was 9.0% in the dapagliflozin group. Although they have a synergistic natriuretic effect along with loop diuretics, this effect seems weaker compared to a combination of loop diuretics and metolazone [35].

**Figure 2:**
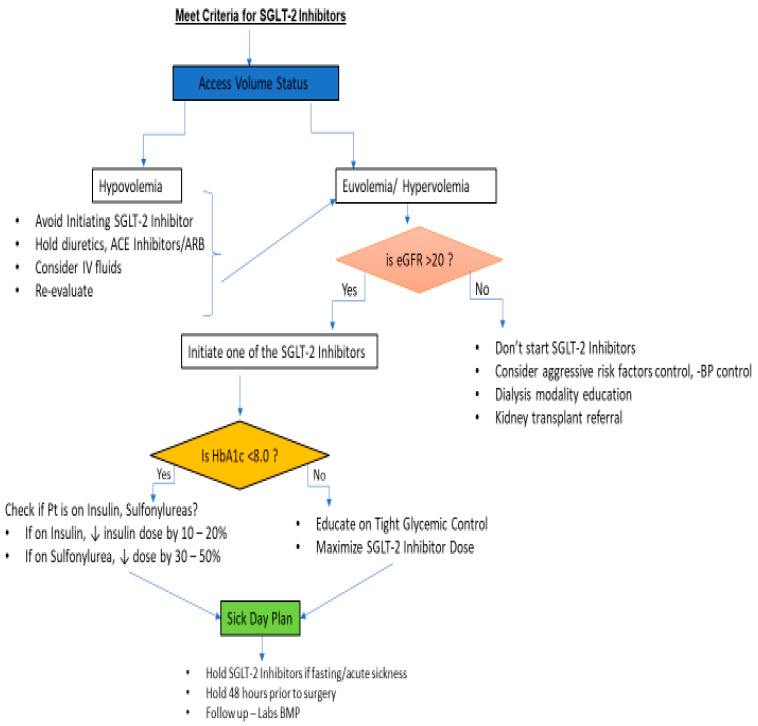
Flowchart for SGLT2 inhibitor use.

In conclusion, the risk of volume depletion should be considered in patients who are already on diuretics therapy, especially the elderly, and those at risk of hypovolemia such as hypotension, fasting, acute sickness, or syncope. It is recommended to assess volume status before initiation of an SGLT-2 inhibitor. Refer to the Figure 2 flowchart.

#### 3.3.6. Adjustment and Interaction with Other Medications

Mineralocorticoid Receptor Antagonists (MRAs) (Table 2): Mineralocorticoid receptor activation due to the upregulation of renin and angiotensin axis causes activation of inflammatory factors and fibrosis, which contribute to the progression of chronic kidney disease as well as congestive heart failure [36].

A new nonsteroidal MRA, finerenone, has been shown to improve proteinuria and blood pressure control in these patients. The FIDELIO-DKD trial included patients with type 2 diabetes mellitus with albuminuria and chronic kidney disease, and these patients were on an ACE inhibitor/ARB at baseline. It found that finerenone decreased the incidence of the primary outcome in terms of the decline of the eGFR by 40% or more by 27%. No significant impact on all-cause mortality or progression into ESKD was noted. It has also been associated with an increased risk of hyperkalemia [37] Tab.

In these trials, most patients were not on a combination of SGLT-2 inhibitor and finerenone, so further data are needed to see if this combination provides any additional benefit [37].

However, in the DAPA-CKD trial, it was reported that 229 out of 4304 patients were receiving MRAs, and this number was too small to determine any significant benefit from this combination. Nonetheless, finerenone is a great addition to the list of medications available for the management of diabetic kidney disease to improve outcomes.

The Kidney Disease Improving Global Outcomes (KDIGO) 2022 guideline as well as the American Diabetes Association 2022 guideline advise using ≥30 mg/day finerenone in patients with albuminuria despite being on a combination of ACE inhibitor and SGLT-2 inhibitor [38].

#### 3.3.7. GLP-1 Agonist

In multiple trials, GLP-1 agonists have proven their efficacy in cardiovascular and kidney protective benefits [39]. The AWARD-7 trial included patients with CKD stages III and IV, which showed improved kidney outcomes with a higher dose of dulaglutide 1.5 mg subcutaneously weekly [40]. Similarly, liraglutide has been shown to improve kidney outcomes, and reduce the progression to ESKD and renal death in patients with diabetic kidney disease [41].

In clinical practice, if the desired glycemic control is not achieved despite being on first-line glucose-lowering therapy and SGLT-2 inhibitor, we recommend adding the GLP-1 agonist due to its cardio- and reno-protective benefit.

Avoid combining GLP-1 agonist with dipeptidyl peptidase 4 (DPP-4) inhibitors as there are no clear-cut data yet available that DPP inhibitors improve outcomes in cardiovascular disease or chronic kidney disease.

## 4. Discussion

SGLT-2 inhibitors have caused a paradigm shift in the management of diabetic kidney disease. The most recent EMPA-KIDNEY trial has confirmed that these agents are not only beneficial in slowing down the progression of underlying kidney disease, including non-diabetes chronic kidney disease, but also benefit patients with low levels of albuminuria and those with an eGFR as low as 20 mL/min/1.73 m^2^ [19].

DAPA-CKD has shown that dapagliflozin can slow down the progression of CKD, regardless of the etiology of CKD, whether it is diabetic kidney disease, hypertensive kidney disease, or glomerulonephritis. It also showed that the protective benefit increases with increased albuminuria and increased hemoglobin A1c, and its effects were also consistent over the spectrum of various eGFR values. SGLT-2 inhibitors provided more absolute benefit in terms of protection for patients who are at the highest absolute risk, including those patients with a low eGFR, high proteinuria, and elevated HbA1c [17,18].

Even regarding heart health, patients at the highest risk of cardiovascular events derive the maximum benefit from these medications. In trials focusing on heart failure and cardiac outcomes, SGLT-2 inhibitors were shown to be beneficial for secondary cardiac prevention. However, recent trials with chronic kidney disease patients indicated that they are also efficacious for the primary prevention of cardiovascular disease. This again confirms that chronic kidney disease should be considered a cardiac risk equivalent [42].

Based on this information, current clinical indications for SGLT-2 inhibitor therapy have been expanded to include patients with or without diabetes mellitus and chronic kidney disease, chronic kidney disease with or without albuminuria, congestive heart failure regardless of diabetes, and diabetes mellitus and congestive heart failure with preserved or low ejection fraction [38,43].

These agents also have a favorable safety profile and should be strongly considered for their protective benefits in patients with chronic kidney disease, atherosclerotic cardiac disease, and heart failure. There is overwhelming evidence showing the effectiveness of these agents in cardiac and kidney protection, and it may not be ethically feasible to carry out additional randomized controlled trials on patients in these groups using SGLT-2 inhibitors.

These data have been incorporated into the guidelines of major medical societies, replacing metformin as the first line of treatment for patients with DM. The American Diabetes Association recommended SGLT-2 inhibitors for organ protection in patients with diabetes mellitus type 2 irrespective of their hemoglobin A1c [44]. The KDIGO Clinical Practice Guideline 2022 for the management of diabetes in chronic kidney disease now includes SGLT-2 inhibitors in addition to metformin, ACE/ARB, and statin as a first-line treatment for T2DM. According to the European Society of Cardiology 2019 guidelines and the European Association for the Study of Diabetes 2019, patients with type 2 diabetes who have ASCVD or high cardiovascular risk should use SGLT-2 inhibitors or GLP-1 receptor agonists [38,43].

### Key Points for Clinical Practice

In clinical practice, we should anticipate around a 20–30% drop in the eGFR after starting an SGLT-2 inhibitor, which does not represent an acute kidney injury [45]. Actually, SGLT-2 inhibitors protect from acute kidney injury [46].

This transient decrease in the eGFR has been attributed to an improvement in intraglomerular hypertension, which in turn leads to reno-protection. This is a reversible decrease in the eGFR and should not prompt clinicians to discontinue therapy until the drop in the eGFR is greater than 30%

The DAPA-CKD trial showed that dapagliflozin can be started with an eGFR as low as 25 mL/min/1.73 m^2^ [17,18]. Subsequently, in 2022, the EMPA-KIDNEY trial confirmed that empagliflozin could be utilized without any safety concerns, even when eGFR levels are as low as 20 mL/min/1.73 m^2^ [19]. Recent KDIGO 2022 guidelines have also incorporated this and now recommend starting an SGLT-2 inhibitor with an eGFR greater than 20 mL/min/1.73 m^2^ [38]. Upon initiation, SGLT-2 inhibitors can be continued until dialysis is started. Avoid use in dialysis patients as studies have not validated their use and lack safety data in dialysis patients.

An attempt should be made to maximize ACE inhibitor/ARB therapy, but this should not delay the introduction of SGLT-2 inhibitors.

Finally, the “Sick day” plan should be discussed with patients clearly, and they should be counseled to withhold SGLT-2 inhibitors if fasting, acutely sick, hypovolemic, or hypotensive. Refer to Figure 2.

## 5. Conclusions

SGLT-2 inhibitors have come a long way from glucose-lowering medication to first-line therapy for cardiac and renal protection in CKD and CHF patients. They have assumed a central role in DKD and cardiovascular management, and their efficacy is beyond doubt. Therefore, clinicians should consider including SGLT-2 inhibitors in their treatment plans for patients with CKD and CHF regardless of diabetes status to ensure optimal outcomes.

Their indications are expanding, and more research is needed to evaluate their efficacy in patients with CKD due to ADPKD, tubulointerstitial nephritis, and those on dialysis/transplant and immunosuppression therapy for stroke prevention.

The following is a list of a few ongoing trials: the RENAL LIFECYCLES, to study the effects of dapagliflozin on dialysis or transplant patients; the PRESERVE trial, to study the effect of dapagliflozin on peritoneal glucose uptake and ultrafiltration in PD patients; and the CONFIDENCE trial, to study the combination of finerenone and empagliflozin.

SGLT-2 inhibitors have filled a large void in the management of DKD. Nonetheless, there is still room for further improvement. New agents such as finerenone, selective endothelin A receptor antagonists, and GLP-1 agonists have been added to the treatment arsenal and should be considered as additional options. However, further studies are required to evaluate the efficacy of these agents in non-diabetic individuals as well as any additional benefit noted in combination with SGLT-2 inhibitors.

## Figures and Tables

**Table 1:** Summary of major clinical Trials.

Trial	CREDENCE	DAPA-HF	DAPA CKD	VERTIS	EMPA KIDNEY
Drug dose	Canagliflozin 100 mg 100 mg	Dapagliflozin 10 mg	Dapagliflozin 10 mg	Ertugliflozin 5, 15 mg	Empagliflozin 10 mg
Year published	June 2019	November 2019	March 2020	June 2020	November 2022
Median follow up	2.6 yrs.	18.2 months	2.4 yrs.	3.5 yrs.	2 yrs.
eGFR on enrollment	>30, UACR 300–5000 mg/gm	>30 (40% pt had eGFR < 60)	25–75, UACR 200–5000 mg/gm	>30	20–45 or >45 < 90, if UACR > 200 mg/gm
DM-2 on enrollment	DM-2 only	42% had DM-2	30% non-diabetic	DM only	Both Diabetic and non-Diabetic 2.2% had DM-type 1
CVD on enrollment	50% participants	All participants	37% participants	76.3% had CAD, 23% had CHF	26% participants
Primary outcome	ESKD, doubling of serum creatinine, death from renal or CV causes.	CV death, heart failure hospitalization, all-cause mortality	ESKD, >50% drop in eGFR, death from renal and CV causes	MACE (CV death, MI, stroke)	ESKD, sustained decrease in eGFR < 10, in eGFR > 40%, death due to renal or cv Causes.
Secondary outcome	Cardiovascular mortality, all-cause mortality, MI, stroke.	ESKD, >50% drop in eGFR lasting >28 days, death from renal causes.	Same as above.	Composite of renal death, dialysis, doubling of serum creatinine	Heart failure hospitalization, all cause mortality, all cause hospitalization
Results	Reduction in primary outcome by 30–35%	Reduction in - CV death, HF by 24%, - All cause mortality by 17%	Reduction in primary outcome by 35–45%	no statistically significant improvement in primary outcomes.	Reduction in primary outcome by 25–30%
NNT (for primary outcome)	22	21	19	Not available	Not available

DM-2—Type 2 Diabetes mellitus; CAD—coronary artery disease; CHF—congestive heart failure; CVD—cardiovascular disease; ESKD—end-stage kidney disease; MI—myocardial infarction; NNT—number needed to treat; UACR—urinary albumin-to-creatinine ratio.

**Table 2:** Dose adjustment in CKD and hepatic dysfunction.

CKD Staging	Stage 3b (eGFR 30–44)	Stage 4 (eGFR 15–29)	Stage 5 (eGFR < 15)	Hepatic Dysfunction
Canagliflozin	100 mg daily	Do not initiate if eGFR < 45		Avoid in Severe impairment (Child-Pugh class C)
Dapagliflozin	10 mg daily	Do not initiate if eGFR < 25. If previously taking, may continue until dialysis		No dosage adjustment Use caution if initiating in severe impairment
Empagliflozin	10, 25 mg daily	- No dosage adjustment necessary for eGFR ≥ 20 US manufacturer does not recommend use for glycemic control for eGFR < 30		No dosage adjustment necessary
Ertugliflozin	Maximum of 15 mg daily, Do not initiate if eGFR < 45	Not recommended		Use is not recommended with Severe impairment (Child-Pugh class C)

## Data Availability

Data sharing not applicable.

## Abbreviations

ASCVD	atherosclerotic cardiovascular disease
ACE-I	angiotensin-converting enzyme inhibitor
ARB	angiotensin receptor blocker
BMP	basic metabolic panel
CHF	congestive heart failure
CKD	chronic kidney disease
DKA	diabetic ketoacidosis
DKD	diabetic kidney disease
ESKD	end-stage kidney disease
eGFR	estimated glomerular filtration rate
ENaC	epithelial sodium channel
GLP-1	glucagon-like peptide 1
HTN	hypertension
MI	myocardial infarction
MR	mineralocorticoid receptor
MRA	mineralocorticoid receptor antagonist
RAAS	renin–angiotensin–aldosterone system
SGLT2	sodium-glucose transport protein 2
T2DM	type II diabetes mellitus
UACR	urine albumin-to-creatinine ratio
